# A freedom from disease study: Schmallenberg virus in the south of England in 2015

**DOI:** 10.1136/vr.103903

**Published:** 2016-09-21

**Authors:** Jessica Eleanor Stokes, Matthew Baylis, Jennifer Sarah Duncan

**Affiliations:** 1 Department of Epidemiology and Population Health, Institute of Infection and Global Health, University of Liverpool, Leahurst Campus, Chester High Road, Neston CH64 7TE, UK; 2 School of Veterinary Science, University of Liverpool, Leahurst Campus, Chester High Road, Neston CH64 7TE, UK

**Keywords:** Arthropod-borne infections (arboviruses), Disease surveillance, Epidemiology, Sheep, Schmallenberg virus, <i>Culicoides</i>

## Abstract

In 2011–2012, northern European livestock faced a threat from a newly emerged virus, Schmallenberg virus (SBV), only a few years after a major outbreak of bluetongue serotype 8 (BTV-8). Like BTV-8, SBV is transmitted by *Culicoides* biting midges to ruminants and spread throughout Europe. SBV, however, spread faster, reaching the UK within three months of initial discovery. Adult ruminants show only mild, if any, clinical signs; however, infection of naive ruminants by SBV during the vulnerable period of gestation leads to abortions, stillbirths and fetal malformations. Although some data exist for the prevalence of SBV on UK sheep farms early in the outbreak, we have no information on its current status. Is SBV still circulating in the UK? To answer this, the authors designed a freedom from disease study across the southernmost counties of the UK. During autumn 2015, 1444 sheep, from 131 farms, were tested for antibodies against SBV by ELISA; 5 samples from 4 farms were twice found positive by ELISA but were later confirmed negative by virus neutralisation test. As the sheep were born between October 2014 and April 2015, the authors conclude that it is unlikely that SBV is still circulating in the south of England.

In November 2011, a novel Orthobunyavirus, of the Simbu serogroup, was identified by metagenomic analysis of cattle presenting with diarrhoea, hyperthermia and reduced milk yield in Germany (Hoffmann and others 2012). The virus was subsequently named Schmallenberg virus (SBV), after the geographic origin of the samples tested. SBV spread rapidly, reaching England within three months of initial outbreak, with the southernmost counties of England all reporting outbreaks of SBV between 2012 and 2013 (EFSA 2012). Like several viruses of the Simbu serogroup and the unrelated bluetongue virus serotype 8 (BTV-8), SBV is transmitted by *Culicoides* biting midges. It is thought that the initial incursion into the UK was via wind dispersal of SBV-infected *Culicoides* from France 113 days before the first report of a malformed lamb (Elbers and others 2013, Sedda and Rogers 2013).

Since its initial discovery, SBV has been detected throughout Europe (EFSA 2014) in domestic cattle, sheep, goats and numerous species of wild ruminants, including camelids. Recently, a high frequency of samples from hunted wild boar in Germany were found to have SBV-specific antibodies (collected 2011/2012) (Mouchantat and others 2015). Additionally, there is a single report of SBV-specific antibodies in a dog, but other studies have failed to find evidence of infection in carnivores (Wensman and others 2013, Mouchantat and others 2015). European studies, conducted in 2011, 2012 and 2013, found animal-level prevalence to range between 8 to 100 per cent and 8.5 to 93.3 per cent in cattle and sheep, respectively (Elbers and others 2012, Gache and others 2013, Nanjiani and others 2013). Herd-level prevalence of UK sheep in 2012/2013 was found to range between 40 and 90 per cent (Nanjiani and others 2013).

SBV infections of adult ruminants are generally asymptomatic; however, if infection of a naive pregnant animal coincides with the vulnerable period of gestation, transmission across the placenta can result in abortions, stillbirths and fetal malformations (Beer and others 2013, Doceul and others 2013). Studies on the related Akabane virus estimate the vulnerable period to be between days 28 and 56 of pregnancy; however, a recent study demonstrated high placental colonisation of SBV when infected at days 45 or 60 of gestation, but a lack of subsequent abortions and malformations observed in the lambs (EFSA 2012, Martinelle and others 2015). Fetal or neonate malformations typically present as arthrogryposis, scoliosis, kyphosis, severe torticollis, brachygnathia and hypoplasia of the central nervous system (Doceul and others 2013). The hypoplasia may be mild to severe, resulting in microencephaly, hydranencephaly and spinal cord and cerebellar hypoplasia (van den Brom and others 2012, Doceul and others 2013). Behavioural and/or neurological disorders are also frequently noted, with lung hypoplasia sometimes observed (Lievaart-Peterson and others 2012). In the case of twins, it is possible for only one to present with malformations, while the other remains viable, or for one twin to present with arthrogryposis, whereas the other presents neurologically (Doceul and others 2013).

A recent study on the duration of immunity in experimentally infected adult sheep has demonstrated SBV-specific IgG antibodies detectable for over one year after a single challenge with SBV (Poskin and others 2015). Additional evidence exists of acquired immunity against reinfection in naturally infected sheep, as well as evidence of maternally derived antibodies in suckling lambs (Rodríguez-Prieto and others 2014). While experimentally infected cattle have been demonstrated to remain immune to reinfection for at least 56 days (Wernike and others 2013).

Four cases of SBV were confirmed on January 16, 2012 (Harris and others 2014). Voluntary reporting recorded 81 and 87 serologically confirmed cases in UK sheep in 2012 and 2013, respectively (AHVLA 2013); however, no cases of SBV were confirmed by PCR in lambs or calves presenting with arthrogryposis by the Animal and Plant Health Agency (APHA) in 2014 or 2015 (APHA, personal communication). A recent study of naive cattle from the Netherlands detected a low level of SBV (<1 per cent) in 2013 (Veldhuis and others 2015). A German study reported a recurrence of SBV in cattle in 2014, despite an apparent decrease in cases the previous year (Wernike others 2015).

The high circulation of SBV in the UK in 2012 and 2013 followed by a subsequent decline in cases in 2014 and 2015 leads to the following question; is this apparent decline in cases in the UK a true decrease in circulation or a lack of reporting? This study aimed to determine whether SBV was still circulating in the southernmost counties of England in 2015 by examining the serological status of sheep born after the 2014 vector period.

## Materials and methods

All animal work was reviewed and approved by the University of Liverpool internal ethics committee (VREC310) and carried out under a Home Office Project Licence (PPL 70/8529). All farmers gave informed written consent and were reminded of their right to withdraw from the study at any point.

**FIG 1:**
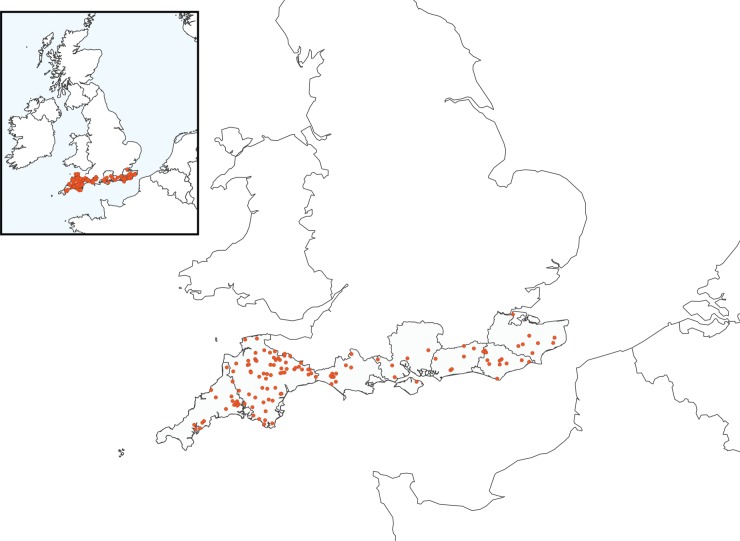
Map of the south of England showing the distribution of sampled farms. Exact farm location has been jittered and enlarged to prevent individual participant identification. Ten farmers asked that their farm location was not mapped

To calculate the number of farms needed to substantiate a prevalence of 2.5 per cent or below the software package, FFD was implemented in *R* (Kopacka 2011). As sheep occur within flocks, a two-stage cluster analysis was used to estimate both the number of flocks and the number of sheep within each flock to be sampled; individual sampling was selected to allow the test sensitivity to remain the same across flocks. The α-error threshold was set to 0.05 (5 per cent). An intraherd prevalence of 20 per cent was set, which is lower than the prevalence recorded in several large-scale continental studies, but closer to the lower range reported in a 2013 UK study (Elbers and others 2012, Nanjiani and others 2013, Veldhuis and others 2013, Méroc and others 2013b); herd sensitivity of Se_herd_=90% was set (EFSA 2014), with a known test sensitivity of Se_2_=97.2% (Bréard and others 2013). The total number of sheep holdings in the southernmost counties of England was extracted from the Department for Environment, Food & Rural Affairs (DEFRA) 2010 census: a total of 6495 sheep holdings were registered. This determined a necessary sample size of 11 sheep per holding collected from 131 holdings to detect prevalence below 2.5 per cent with 95 per cent confidence. Holdings were recruited for the study through the National Sheep Association (NSA) South West show, NSA magazine and large animal veterinary practices (Fig 1). The number of holdings sampled per county was stratified based on DEFRA 2010 census data: Cornwall (n=18), Devon (n=67), Dorset (n=12), Hampshire (n=5), Sussex (n=20) and Kent (n=9).

Blood samples were collected between September 15, 2015, and December 11, 2015, from the jugular vein of 12 sheep per holding (11, plus 1 to account for failures). Sampling began after the spring and summer peaks in midge activity, with the majority of samples collected after the final autumn peak in midge activity (Sanders and others 2011). All sampled sheep were born after October 2014 and were more than six months old at time of sampling to exclude animals with immunity following infection in 2012, 2013 or 2014 and to avoid maternal antibodies. This assumption is based on the maternal antibodies of calves lasting less than six months for both SBV and Akabane virus (Tsutsui and others 2009, Elbers and others 2014). Serum was extracted from the blood samples and analysed by a commercially available SBV antibody ELISA (ID Screen SBV indirect, IDvet, France) as per the manufacturer's instructions. Serum samples were considered negative if the S/P% (sample to positive percentage) was up to 50 per cent calculated as per manufacturer's instructions. Samples returning an S/P% greater than 50 per cent were sent to the APHA to be confirmed by a virus neutralisation test (VNT). An equal number of samples returning an S/P% less than 50 per cent were also sent as blind controls; they were selected randomly using the RANDBETWEEN function in Microsoft Excel to determine farm number and then sample number from farm. Two positive controls were used in the VNT, with VNT titres of 1/40 and 1/80, respectively. Samples were determined to be negative if the VNT titre was greater than 1/5 based on the minimum dilution undertaken at the APHA (La Rocca, personal communication).

## Results

A total of 1572 sheep from 131 holdings were sampled between September 15 and December 11, 2015. Flock sizes ranged from 20 to 5000, all sheep sampled were born between October 2014 and April 2015. Of the 131 holdings sampled, 103 were lowland flocks, 8 hill, 8 upland, 10 had flocks across lowland, hill and/or upland pastures and 2 holdings declined to answer or were unsure.

Half (50.0 per cent) of farmers (57/124, seven farmers declined to answer) reported that they had previously suspected cases of SBV infection in their flocks in the form of birth of lambs showing typical congenital abnormalities. Of these 57 farmers, 12 had cases that were diagnosed by a vet but not laboratory confirmed, while 13 had cases that were diagnosed by a vet and laboratory confirmed as SBV. In the remaining 32 suspect case farms, none had disease diagnosed, either by a vet or laboratory.

Only 13.7 per cent (17/124) of farmers stated that they had vaccinated their sheep against SBV; 15 farmers stated they vaccinated in 2013, while 2 farmers vaccinated in both 2013 and 2014. One farmer vaccinated only their cattle against SBV but not their sheep.

By contrast, only 1.6 per cent (2/124) of the farmers stated that they had had cases of BTV on farm, with 78.2 per cent (97/124) stating that they had vaccinated against BTV for at least one year.

A total of 11 sheep from each holding were tested by ELISA for antibodies against SBV (1444 samples in total). Overall nine samples, from eight holdings, returned doubtful or positive (S/P per cent > 50 per cent) results for antibodies to SBV when tested by ELISA. These samples were retested by ELISA, with five samples, from four holdings returning positive for antibodies against SBV. No antibodies were detected in these five samples when tested by VNT at the APHA (Table 1).

**TABLE 1:** Schmallenberg virus (SBV) ELISA and virus neutralisation test (VNT) results in samples that returned a positive ELISA test result, holding ID, county of farm, breed of sheep, birth and sample dates, ELISA titre (S/P%), VNT result, previous self-reporting of suspected cases on farm by farmer and if the farm vaccinated against SBV in 2013

Sheep ID	Holding ID	County	Breed	Born	Sampled	S/P% ELISA	S/P% ELISA retest	VNT result	Previous Suspected SBV cases on farm?	Vaccinated flock against SBV in 2013
1	21	Dorset	Texel	March 2015	September 2015	166.55*	9.82†	Not Tested	No	Yes
2	25	Dorset	Poll Dorset	January 2015	September 2015	64.93*	46.40†	Not Tested	No	No
3	46	Hampshire	Hampshire	December 2014	October 2015	60.77*	44.41†	Not Tested	Yes‡	No
4	48	Cornwall	Roussin	February 2015	October 2015	55.43*	39.54†	Not Tested	Yes	No
5	59	Devon	Highlander	March 2015	October 2015	78.14*	120.62*	Negative	No	No
6	108	Sussex	Dorset	February 2015	November 2015	110.32*	80.97*	Negative	Yes‡	No
7	113	Sussex	Charolais	February 2015	November 2015	90.61*	127.66*	Negative	Yes	No
8	113	Sussex	Charolais	February 2015	November 2015	66.45*	125.91*	Negative		
9	121	Cornwall	Lleyn X Texel	March 2015	November 2015	117.97*	123.79*	Negative	No	No
10	12	Devon	Poll Dorset	December 2014	September 2015	3.19†	Not Tested	Negative	Yes	No
11	50	Cornwall	Zwartble	March 2015	October 2015	3.93†	Not Tested	Negative	No	No
12	99	Kent	Charolais	March 2015	November 2015	8.46†	Not Tested	Negative	Yes	No
13	106	Sussex	Charolais	March 2015	November 2015	2.50†	Not Tested	Negative	Yes‡	No
14	115	Sussex	Southdown X Dorset	April 2015	November 2015	5.18†	Not Tested	Negative	Yes‡	No

Samples 10–14 are negative controls for the VNT

*ELISA positive S/P%

†ELISA negative S/P%

‡Laboratory-confirmed cases of SBV on farm

## Discussion

This study found it unlikely that any antibodies against SBV were circulating in the sheep tested. As these sheep were born between October 2014 and May 2015, we can be 95 per cent confident that if SBV was circulating in the south of England in the 2015 vector period, it was present below the 2.5 per cent prevalence threshold designed by this study. Using a similar testing procedure, a study of cattle in the Netherlands determined a maximum possible prevalence of herds to be <1 per cent prevalence in 2013 (Veldhuis and others 2015).

The specificity of the commercial ELISA kit used was reported to be 99.8 per cent, giving a likely false-positive rate of ∼3 samples of the 1444 tested. Initially 9 out of the 1444 samples returned positive by ELISA for SBV-specific antibodies, higher than the calculated test false-positive rate. However, other studies have cast doubt on the high specificity of the test if the virus is circulating below the peak outbreak levels, with a false-positive rate of 41 per cent reported in wild cervids (Laloy and others 2014) tested by both the indirect ELISA used here and the VNT (Bréard and others 2013, Laloy and others 2014). The use of VNTs as conformational tests for commercial ELISAs is considered advisable due to the high (∼99–100 per cent) sensitivity and specificity of the VNT (Loeffen and others 2012).

As observed during the height of the SBV outbreak in Europe, the transmission of SBV is highly efficient, spreading rapidly both within and between flocks (Veldhuis and others 2013, Méroc and others 2013a, EFSA 2014, Wernike and others 2014). This spread was far faster than that of BTV-8, likely due to the much shorter viraemia, much higher probability of host to vector transmission and SBV's predicted faster replication rate and replication at a lower temperature threshold than BTV-8 (Gubbins and others 2014). Even with low levels of SBV circulation and few susceptible hosts on farm, previous studies have demonstrated eventual seroconversion of these individuals (Elbers and others 2013). These characteristics of SBV make it also highly unlikely that the five ELISA-positive samples were true positives as that would mean SBV was persisting at a very low prevalence, within a large naive population. However, this does not mean that it is impossible for SBV to persist at very low levels, particularly if reintroduced late in the *Culicoides* season, as the current knowledge of the epidemiology of SBV is still expanding.

Despite this, surveillance for SBV should continue, with a German study describing a decline of SBV occurrence in cattle in 2013 compared with 2011–2012 seroprevalence, followed by an increase in cases the following year (Wernike and others 2015). This is a frequent occurrence with midge-borne arboviruses. For example, since the end of the most recent BTV-8 outbreak, the serotype was considered absent from France, with disease-free status granted in 2012; only for it to re-emerge in August 2015 (Sailleau and others 2015). It has been postulated that this new outbreak may have re-emerged from wildlife reservoirs, with red deer in Spain previously testing positive for BTV when local livestock remained disease free (Ruiz-Fons and others 2014). If this was indeed the case, then greater emphasis should be put on surveillance of wild ruminant populations to determine freedom within this potential reservoir source, particularly as far more wild species have been demonstrated to have SBV-specific antibodies, with far higher prevalence in populations described, than for BTV-8 (Rossi and others 2015). An alternative to invasive on-farm procedures would be the widespread trapping of *Culicoides* for surveillance, perhaps by bulk testing by county/canton to rapidly test large numbers of the insects (Poskin and others 2016). Targeted surveillance could also be used, collecting *Culicoides* at sites deemed ‘high risk’ for possible passive wind transfer from Europe, particularly in the event of recurrence on the continent.

Regardless of the current status of SBV in Europe, this study has highlighted a large, naive population, susceptible to future potential outbreaks within the south of England. Effective surveillance systems are therefore needed to warn vets and farmers of future disease risks.

## References

[R1] AHVLA, 2013. Schmallenberg virus- Updated testing results. http://www.farminguk.com/content/knowledge/20130424sbv-statistics.pdf (Accessed 2 Jun 2015)

[R2] BEER M., CONRATHS F. J. & VAN DER POEL W. H. M. (2013) “Schmallenberg virus”—a novel orthobunyavirus emerging in Europe. Epidemiology and Infection 141, 1–8 10.1017/S0950268812002245 23046921 PMC9152057

[R3] BRÉARD E., LARA E., COMTET L., VIAROUGE C., DOCEUL V., DESPRAT A., VITOUR D., POZZI N., CAY A.B., DE REGGE N., POURQUIER P., SCHIRRMEIER H., HOFFMANN B., BEER M., SAILLEAU C. & ZIENTARA S. (2013) Validation of a commercially available indirect ELISA using a nucleocapside recombinant protein for detection of Schmallenberg virus antibodies. PLoS ONE 8, e53446 10.1371/journal.pone.0053446 23335964 PMC3546048

[R4] DOCEUL V., LARA E., SAILLEAU C., BELBIS G., RICHARDSON J., BRÉARD E., VIAROUGE C., DOMINGUEZ M., HENDRIKX P., CALAVAS D., DESPRAT A., LANGUILLE J., COMTET L., POURQUIER P., ELÉOUËT J.-F., DELMAS B., MARIANNEAU P., VITOUR D. & ZIENTARA S. (2013) Epidemiology, molecular virology and diagnostics of Schmallenberg virus, an emerging orthobunyavirus in Europe. Veterinary Research 44, 31 10.1186/1297-9716-44-31 23675914 PMC3663787

[R5] EFSA (2012) EFSA - Scientific Report of EFSA: “Schmallenberg” virus: analysis of the epidemiological data and Impact assessment. EFSA Journal 10 http://www.efsa.europa.eu/en/efsajournal/pub/2768.htm. Accessed June 2, 2015

[R6] EFSA (2014) Schmallenberg virus: state of Art. EFSA Journal 12, 1–54

[R7] ELBERS A. R. W., LOEFFEN W. L. A., QUAK S., DE BOER-LUIJTZE E., VAN DER SPEK A. N., BOUWSTRA R., MAAS R., SPIERENBURG M. A. H., DE KLUIJVER E. P., VAN SCHAIK G. & VAN DER POEL W. H. M. (2012) Seroprevalence of Schmallenberg virus antibodies among dairy cattle, the Netherlands, winter 2011–2012. Emerging Infectious Diseases 18, 1065–1071 22709656 10.3201/eid1807.120323 PMC3376820

[R8] ELBERS A. R. W., STOCKHOFE-ZURWIEDEN N. & VAN DER POEL W. H. M. (2014) Schmallenberg virus antibody persistence in adult cattle after natural infection and decay of maternal antibodies in calves. BMC Veterinary Research 10, 103 10.1186/1746-6148-10-103 24885026 PMC4013805

[R9] ELBERS A.R. W., MEISWINKEL R., VAN WEEZEP E., KOOI E.A. & VAN DER POEL W. H. M. (2013) Schmallenberg Virus in *Culicoides* Biting Midges in the Netherlands in 2012. Transboundary and Emerging Diseases 62, 339–342 10.1111/tbed.12128 23890155

[R10] GACHE K., DOMINGUEZ M., PELLETIER C., PETIT E., CALAVAS D., HENDRIKX P. & TOURATIER A. (2013) Schmallenberg virus: a seroprevalence survey in cattle and sheep, France, winter 2011–2012. The Veterinary Record 173, 141 10.1136/vr.101377 23804407

[R11] GUBBINS S., TURNER J., BAYLIS M., VAN DER STEDE Y., VAN SCHAIK G., ABRAHANTES J. C. & WILSON A. J. (2014) Inferences about the transmission of Schmallenberg virus within and between farms. Preventive Veterinary Medicine 116, 380–390 10.1016/j.prevetmed.2014.04.011 24857371 PMC4204990

[R12] HARRIS K. A., EGLIN R. D., HAYWARD S., MILNES A., DAVIES I., COOK A. J. C. and DOWNS S. H. (2014) The impact of Schmallenberg virus on British sheep farms during the 2011/2012 lambing season. The Veterinary Record 175, 16–23 10.1136/vr.102295 PMC4145415 24795165

[R13] HOFFMANN B., SCHEUCH M., HÖPER D., JUNGBLUT R., HOLSTEG M., SCHIRRMEIER H., ESCHBAUMER M., GOLLER K. V., WERNIKE K., FISCHER M., BREITHAUPT A., METTENLEITER T.C. & BEER M. (2012) Novel orthobunyavirus in Cattle, Europe, 2011. Emerging Infectious Diseases 18, 469–472 10.3201/eid1803.111905 22376991 PMC3309600

[R14] KOPACKA I. (2011) FFD: Freedom from Disease. R Package Version 1.0.-1. http://cran.r-project.org/package=FFD. Accessed June 28, 2015

[R15] LALOY E., BRÉARD E., SAILLEAU C., VIAROUGE C., DESPRAT A., ZIENTARA S., KLEIN F., HARS J. & ROSSI S. (2014) Schmallenberg virus infection among red deer, France, 2010–2012. Emerging Infectious Diseases 20, 131–134 10.3201/eid2001.130411 24377838 PMC3884713

[R16] LIEVAART-PETERSON K., LUTTIKHOLT S. J. M., VAN DEN BROM R. & VELLEMA P. (2012) Schmallenberg virus infection in small ruminants – First review of the situation and prospects in Northern Europe. Small Ruminant Research 106, 71–76 10.1016/j.smallrumres.2012.03.006

[R17] LOEFFEN W., QUAK S., DE BOER-LUIJTZE E., HULST M., VAN DER POEL W., BOUWSTRA R. & MAAS R. (2012) Development of a virus neutralisation test to detect antibodies against Schmallenberg virus and serological results in suspect and infected herds. Acta veterinaria Scandinavica 54, 44 10.1186/1751-0147-54-44 22871162 PMC3503834

[R18] MARTINELLE L., POSKIN A., DAL POZZO F., DE REGGE N., CAY B. & SAEGERMAN C. (2015) Experimental infection of sheep at 45 and 60 days of gestation with schmallenberg virus readily led to placental colonization without causing congenital malformations. PLoS ONE 10, e0139375 10.1371/journal.pone.0139375 26418420 PMC4587791

[R19] MÉROC E., DE REGGE N., RIOCREUX F., CAIJ A. B., VAN DEN BERG T. & VAN DER STEDE Y. (2013b) Distribution of Schmallenberg Virus and Seroprevalence in Belgian Sheep and Goats. Transboundary and Emerging Diseases 61, 425–431 10.1111/tbed.12050 23305427

[R20] MÉROC E., POSKIN A., VAN LOO H., QUINET C., VAN DRIESSCHE E., DELOOZ L., BEHAEGHEL I., RIOCREUX F., HOOYBERGHS J., DE REGGE N., CAIJ A.B., VAN DEN BERG T. & VAN DER STEDE Y. (2013a) Large-scale cross-sectional serological survey of schmallenberg virus in belgian cattle at the end of the first vector season. Transboundary and Emerging Diseases 60, 4–8 10.1111/tbed.12042 23206240

[R21] MOUCHANTAT S., WERNIKE K., LUTZ W., HOFFMANN B., ULRICH R. G., BÖRNER K., WITTSTATT U. & BEER M. (2015) A broad spectrum screening of Schmallenberg virus antibodies in wildlife animals in Germany. Veterinary Research 46, 469 10.1186/s13567-015-0232-x PMC4579581 26394618

[R22] NANJIANI I. A., AITKEN P. & WILLIAMS P. (2013) Prevalence of seropositive sheep within flocks where Schmallenberg virus infection was suspected or confirmed. The Veterinary Record 173, 371 10.1136/vr.101796 24056995

[R23] POSKIN A., THÉRON L., HANON J.-B., SAEGERMAN C., VERVAEKE M., VAN DER STEDE Y., CAY B. & DE REGGE N. (2016) Reconstruction of the Schmallenberg virus epidemic in Belgium: Complementary use of disease surveillance approaches. Veterinary Microbiology 183, 50–61 10.1016/j.vetmic.2015.11.036 26790935

[R24] POSKIN A., VERITE S., COMTET L., VAN DER STEDE Y., CAY B. & DE REGGE N. (2015) Persistence of the protective immunity and kinetics of the isotype specific antibody response against the viral nucleocapsid protein after experimental Schmallenberg virus infection of sheep. Veterinary Research 46, 119 10.1186/s13567-015-0260-6 26472116 PMC4608186

[R25] RODRÍGUEZ-PRIETO V., KUKIELKA D., MOURIÑO M., PARADELL H., PLAJA L., URNIZA A. & SÁNCHEZ-VIZCAÍNO J. M. (2014) Natural Immunity of Sheep and Lambs Against the Schmallenberg Virus Infection. Transboundary and Emerging Diseases 63, e220–e228 10.1111/tbed.12256 25100663

[R26] ROSSI S., VIAROUGE C., FAURE E., GILOT-FROMONT E., GACHE K., GILBERT P., VERHEYDEN H., HARS J., KLEIN F., MAILLARD D., GAUTHER D., GAME Y., POZET F., SAILLEAU C., GARNIER A., ZIENTARA S. & BREARD E. (2015) Exposure of Wildlife to the Schmallenberg Virus in France (2011–2014): Higher, Faster, Stronger (than Bluetongue)! Transboundary and Emerging Diseases 10.1111/tbed.12371 10.1111/tbed.12371 25958882

[R27] RUIZ-FONS F., SÁNCHEZ-MATAMOROS A., GORTÁZAR C. & SÁNCHEZ-VIZCAÍNO J. M. (2014) The role of wildlife in bluetongue virus maintenance in Europe: lessons learned after the natural infection in Spain. Virus Research 182, 50–58 10.1016/j.virusres.2013.12.031 24394295

[R28] SAILLEAU C., BREARD E., VIAROUGE C., VITOUR D., ROMEY A., GARNIER A., FABLET A., LOWENSKI S., GORNA K., CAIGNARD G., PAGNEUX C. & ZIENTARA S. (2015) Re-emergence of Bluetongue virus serotype 8 in France, 2015. Transboundary and Emerging Diseases 10.1111/tbed.12453 10.1111/tbed.12453 26617414

[R29] SANDERS C. J., SHORTALL C. R., GUBBINS S., BURGIN L., GLOSTER J., HARRINGTON R., REYNOLDS D. R., MELLOR P. S. & CARPENTER S. (2011) Influence of season and meteorological parameters on flight activity of Culicoides biting midges. Journal of Applied Ecology 48, 1355–1364 10.1111/j.1365-2664.2011.02051.x

[R30] SEDDA L. & ROGERS D. J. (2013) The influence of the wind in the Schmallenberg virus outbreak in Europe. Scientific Reports 3, 3361 10.1038/srep03361 24285292 PMC6506448

[R31] TSUTSUI T., YAMAMOTO T., HAYAMA Y., AKIBA Y., NISHIGUCHI A., KOBAYASHI S. & YAMAKAWA M. (2009) Duration of Maternally Derived Antibodies against Akabane Virus in Calves: Survival Analysis. Journal of Veterinary Medical Science 71, 913–918 10.1292/jvms.71.913 19652478

[R32] VAN DEN BROM R., LUTTIKHOLT S. J. M., LIEVAART-PETERSON K., PEPERKAMP N. H. M. T., MARS M. H., VAN DER POEL W. H. M. & VELLEMA P. (2012) Epizootic of ovine congenital malformations associated with Schmallenberg virus infection. Tijdschrift Voor Diergeneeskunde 137, 106–111 22393844

[R33] VELDHUIS A. M. B., MARS M. H., ROOS C. A. J., VAN WUYCKHUISE L. & VAN SCHAIK G. (2015) Two Years After the Schmallenberg Virus Epidemic in the Netherlands: Does the Virus still Circulate? Transboundary and Emerging Diseases 10.1111/tbed.12349 10.1111/tbed.12349 25903767

[R34] VELDHUIS A. M. B., VAN SCHAIK G., VELLEMA P., ELBERS A. R. W., BOUWSTRA R., VAN DER HEIJDEN H. M. J. F. & MARS M. H. (2013) Schmallenberg virus epidemic in the Netherlands: spatiotemporal introduction in 2011 and seroprevalence in ruminants. Preventive Veterinary Medicine 112, 35–47 10.1016/j.prevetmed.2013.06.010 23906391

[R35] WENSMAN J. J., BLOMQVIST G., HJORT M. & HOLST B. S. (2013) Presence of antibodies to Schmallenberg virus in a dog in Sweden. Journal of Clinical Microbiology 51, 2802–2803 10.1128/JCM.00877-13 23740729 PMC3719652

[R36] WERNIKE K., ESCHBAUMER M., SCHIRRMEIER H., BLOHM U., BREITHAUPT A., HOFFMANN B. & BEER M. (2013) Oral exposure, reinfection and cellular immunity to Schmallenberg virus in cattle. Veterinary microbiology 165, 155–159 10.1016/j.vetmic.2013.01.040 23452751

[R37] WERNIKE K., HOFFMANN B., CONRATHS F. J. & BEER M. (2015) Schmallenberg Virus Recurrence, Germany, 2014 - Volume 21, Number 7—July 2015 - Emerging Infectious Disease journal - CDC. http://wwwnc.cdc.gov/eid/article/21/7/15-0180_article. Accessed June 29, 2015 10.3201/eid2107.150180 PMC4480399 26079975

[R38] WERNIKE K., SILAGHI C., NIEDER M., PFEFFER M. & BEER M. (2014) Dynamics of Schmallenberg virus infection within a cattle herd in Germany, 2011. Epidemiology and Infection 142, 1501–1504 10.1017/S0950268813002525 24128891 PMC9151231

